# Latent profile analysis of students’ reading development and the relation of cognitive variables to reading profiles

**DOI:** 10.1007/s11881-020-00196-9

**Published:** 2020-04-14

**Authors:** Leena Holopainen, Nhi Hoang, Arno Koch, Doris Kofler

**Affiliations:** 1 School of Education and Psychology, Philosophical Faculty, University of Eastern Finland, P.O. Box 111, FI-80101 Joensuu, Finland; 2 Instutute for Special and Inclusive Education, Justus Liebig University Giessen, Karl-Glöcknerstr. 21 B, 35394 Giessen, Germany; 3 Faculty of Education, Free University of Bozen, Regensburger Allee 16, 39042 Brixen - Bressanone, Italy

**Keywords:** Latent profiles, Orthography, Phonological awareness, Rapid naming, Reading development,

## Abstract

Previous studies have showed that early problems with word decoding can lead to poor performance in text reading and comprehension and suggest that poor readers often struggle with reading deficits throughout their school years. Therefore, early detection of those children who are at risk for slow reading development and/or who belong to the lowest reading profiles is essential in order to organize proper support. The present study explores the heterogeneity and prevalence of latent reading profiles among 769 Finnish- and German-reading students during their first and second school years in three countries (Finland, Germany, and Italy) using latent profile analysis. The results identified three latent profiles among Finnish readers, one of which (sentence-level reading) was identified as developing slowly. Among German-reading students, four latent profiles were discovered, two of which were identified as developing slowly. The results of ordinal logistic regression modeling show that rapid automatic naming (RAN) was significantly related to poorer reading profiles among Finnish- and German-reading students, and that the poorer results in letter-sound connection testing among the German-reading group was also significantly related to poorer reading profiles. Although the educational systems have some differences between Germany and German-speaking areas of Italy, no significant country effect was detected. In addition, a child’s age and spoken language did not significantly affect the student’s reading profile.

## Introduction

Most cross-linguistic studies of reading development have typically focused on whole-sample averages and correlations, rather than using a person-oriented approach that allows the researcher to analyze heterogeneous profiles that emerge within the samples. Profiling studies are pertinent because the link between reading and reading-related skills across different orthographies is likely to vary between individuals (Davies, Cuetos, & Glez-Seijas, 2007; Landerl & Wimmer, 2008; Lyytinen et al., 2005). A profile that simultaneously examines several different aspects of reading can provide a comprehensive overview of the mechanisms behind the accumulation of risk and can therefore help to identify children at higher risk and encourage the development of proper support systems. In the present study, a person-oriented approach was used to examine the development of children’s reading skills. This study followed a large sample of children from first through second grades in two separate orthographies (German and Finnish) and three different educational systems (Finnish, German, and Italian) in order to better understand which of the factors that affect reading development are universal and which are orthography-specific (Landerl et al., 2013; Seymour, Aro, & Erskine, 2003).

### Orthography and reading difficulties

There is a large body of evidence to suggest that reading difficulties are caused by problems with letter-sound connections, phonological processing, and/or rapid automatic naming (RAN) (Hulme & Snowling, 2009; Hulme, Bowyer-Crane, Carroll, Duff, & Snowling, 2012; Ozernov-Palchik et al., 2017; Schatschneider, Fletcher, Francis, Carlson, & Foorman, 2004; Snellings, van der Leij, Blok, & de Jong, 2010; Vellutino, Fletcher, Snowling, & Scanlon, 2004). Success while learning to read any alphabet requires that the child learn to map letters to their associated phonemes. The relationship between phonological awareness and letter-sound connection is much studied, and several previous studies have demonstrated that the relationship between them is reciprocal (e.g. Mann & Wimmer, 2002; Wagner, Torgesen, & Rashotte, 1994). It is now widely accepted that a phonological core deficit can trigger the cognitive manifestation of reading difficulties (Stanovich, 1988); this deficit is considered an important predictor of poor reading abilities during the early years of formal education (Boets et al., 2010). On the other hand, in a Dutch study by de Jong and van der Leij (2003), and in recent study by Knoop-van Campen, Segers, and Verhoeven (2018), it was shown that children with dyslexia in the upper grades show problems on more difficult phonological tasks, such as phoneme blending or phoneme deletion. This could be caused by the higher demand these tasks put on working memory. Thus, linguistic differences may play a role in the relation between phonological awareness and decoding. This relation is stronger in opaque languages than in more transparent orthographies (Georgiou, Parrila, & Papadopoulos, 2008; Landerl & Wimmer, 2008).

RAN has been associated with a number of different cognitive components related to reading difficulties; for example, reading difficulties can be characterized as single deficits in phonological awareness or RAN, or as double-deficits in both phonological awareness and RAN (Kirby, Parrila, & Pfeiffer, 2003; O'Brien, Wolf, & Lovett, 2012; Papadopoulos, Georgiou, & Kendeou, 2009). Letter knowledge has also been found to predict performance on RAN tasks (Schatschneider et al., 2004), while RAN has been found to predict reading skills after controlling for letter knowledge (Landerl & Wimmer, 2008; Lervåg, Bråten, & Hulme, 2009). Currently, there appears to be no clear evidence to support a single prevailing explanation for the RAN-reading relationship (Poulsen, Juul, & Elbro, 2015).

Even within alphabetic orthographies, there are differences in the consistency of correspondence between symbols and sounds. The orthographic depth hypothesis (Katz & Frost, 1992) suggests that readers of transparent orthographies (e.g., German, Finnish) are more likely to experience reading success through using letter-sound mapping than are readers of opaque orthographies (e.g., English, French). In addition, different lexical, phonological, and structural factors all contribute to explaining cross-language differences in reading acquisition by children, as has been introduced in the psycholinguistic grain size theory (Goswami, 2010; Ziegler & Goswami, 2005). Among opaque orthographies, reading difficulties are characterized by poor word-level reading accuracy, as well as by problems with reading speed (Ziegler, Perry, Ma-Wyatt, Ladner, & Schulte-Körne, 2003) rather than in reading inaccuracy (Holopainen, Ahonen, & Lyytinen, 2001; Wimmer, Mayringer, & Landerl, 1998; Ziegler et al., 2003). For example, in Finnish, with consistent spelling and pronunciation from graphemes to phonemes and from phonemes to graphemes, the average child’s ability to accurately read words is very high by the middle of first grade (Aro, 2017; Seymour et al., 2003), but slow reading seems to be a persistent handicap (Aro et al., 2018; Lyytinen, Shu, & Richardson, 2015). German-speaking children who experience reading difficulties generally demonstrate problems with reading accuracy only during the early phases of reading acquisition (Wimmer, 1996) and show slow, accurate word-level reading after a few years of schooling (Landerl, Wimmer, & Frith, 1997). Although German is also regarded as a transparent orthography, phoneme-grapheme correspondence is often inconsistent, which can affect the development of proper spelling abilities (Ise & Schulte-Körne, 2010; Ziegler & Goswami, 2005).

One possible explanation for cross-language differences in reading difficulties is that phonologically consistent orthography can help children, even those with phonological deficits, to understand the alphabetic principles and phonemic structures of speech (Landerl, 2003). Children who find the reading process difficult, for example, due to an underlying deficit in phonological processing, tend to receive consistently positive feedback, as reading almost always leads to the correct output. However, the process of phonological decoding remains slow and extremely laborious for children who experience reading difficulties, and this process can be interpreted as slow reading fluency. Some studies have found that children who exhibit slow reading fluency also display a reduced ability to name familiar symbols, shown, e.g., in RAN tasks (Georgiou, Torppa, Manolitsis, Lyytinen, & Parrila, 2012; Moll & Landerl, 2009; Torppa, Soodla, Lerkkanen, & Kikas, 2019).

As multiple components are necessary for children to become successful readers, reading difficulties can be determined by factors that contribute to, or reflect, deficits in reading and reading comprehension (Baker & Beall, 2008; Klicpera & Gasteiger-Klicpera, 1995). The ability to read fluently is a critical bridge between decoding and comprehension (Rasinski, 2004). At the word level, good and poor readers seem to differ in their knowledge of vocabulary as well as in their decoding skills, while at the sentence level, poor readers tend to exhibit less syntactic knowledge and have more problems creating a locally coherent representation of related sentences (Cain, 2009).

### Profiling Reading development

It is important to recognize differences in performance between average and below-average reading groups as early as possible. Findings have suggested that early identification of reading difficulties is possible and, just as importantly, that “one-size-fits-all” interventions are likely to be less effective at supporting different risk profiles (Allor, Champlin, Gifford, & Mathes, 2010; Ozernov-Palchik et al., 2017; Vaughn & Fletcher, 2012).

Some previous researchers have used cluster analysis, latent profile analysis (LPA), or latent class analysis (LCA) to ascertain person-oriented reading profiles or groups among beginning readers. In addition, some studies have focused on whether a particular measurement (e.g., phonological awareness) taken at one point during a child’s reading development may be correlated with the reading outcomes of different groups (Scarborough, 1998). In one longitudinal study, LCA was used to identify distinct subtypes of reading development among a large sample of Finnish children who were studying in general education and who were tested twice per year in the first and second grades (Torppa et al., 2007). Based on the children’s performance on tests for single-word identification, reading fluency, and reading comprehension, five reading groups were identified: (1) poor readers, (2) slow decoders, (3) poor comprehenders, (4) average readers, and (5) good readers. In a similar Swedish study (Wolff, 2010), LPA was used to characterize a sample of 9-year-old children through several reading measures (e.g., reading texts and graphs, word reading, and reading fluency). Nine reader profiles were identified based on the results of this study: (1) very good readers, (2) average readers, (3) poor graph readers, (4) hyperlexic readers, (5) garden-variety poor readers, (6) below-average decoders, (7) below-average fluency, (8) low comprehension, and (9) poor decoders (Wolff, 2010).

A recent study of English-speaking students (Grimm, Solari, McIntyre, & Denton, 2018) revealed the latent profiles of at-risk and not at-risk first-grade readers. Latent profile analyses were conducted for each sub-group to measure phonological awareness, decoding, linguistic comprehension, and oral reading fluency. The analyses showed two latent profiles of at-risk students and three latent profiles of not at-risk students. Phonological awareness and decoding measures were best at differentiating latent profiles, although linguistic comprehension was an important variable in order to differentiate the lowest-performing students. Ozernov-Palchik et al. (2017) analyzed a large group of English-speaking pre-kindergarten and kindergarten students using LPA and studied the longitudinal stability of the latent class groups that were identified among kindergarten children in order to recognize at-risk and not at-risk first-grade readers. Six distinct reading profiles emerged from this analysis: (1) average performers, (2) high performers, (3) below-average performers, (4) RAN risk, (5) PA risk, and (6) double-deficit risk. Each of these reading profiles revealed a 100% longitudinal stability of class membership (Ozernov-Palchik et al., 2017).

### Instruction and reading development

Reading difficulties have also been associated with environmental factors, such as methods in reading instruction (Johnston, McGeown, & Watson, 2011; McGeown, Johnston, & Medford, 2012) and the educational system (e.g., pre-primary education, child’s age when they began school, etc.) (Sellès et al., 2015). There is evidence to suggest that the methods used to teach children how to read can influence their skills and predict their reading success and the ways in which their reading development will continue (Connelly, Thompson, Fletcher-Flinn, & McKay, 2009).

#### Finland

In the Finnish educational system, children begin formal schooling at the age of seven, following an obligatory 1 year of pre-primary education for 6-year-old children. It is important to note that the Finnish pre-primary curriculum does not include direct teaching of reading and spelling, although teachers do usually introduce letters in a play-like fashion. The pre-primary education curriculum creates a foundation for literacy skills by, for example, encouraging children to practice phonological awareness skills. Finnish teachers usually use synthetic (phonetic) reading instruction in the first grade, as did all teachers who participated in this study. Synthetic reading instruction encourages children to begin systematically practicing letter names, letter-sound connections, and syllable decoding, with a focus on teaching children to decode and form words from syllables. As decoding skills become automatic during the fall term in the first grade and continue in the second grade, reading instruction begins to focus more on practicing sentence-level reading, reading fluency, and reading comprehension (Finnish National Board of Education, 2016). The average Finnish class size is approximately 16 students. In Finland, special needs education is primarily provided within mainstream schools by part-time special education teachers.

#### Germany

In the Hessen area of Germany, where this portion of the research was completed, school attendance is obligatory after the age of six. In Germany, kindergarten is an early childhood educational institution attended by children aged three to 6 years and is considered an integral part of German childhood and a formative part of early education. Kindergarten can also include a year of preschool, where basic competencies are developed in different fields of education (e.g., music, art, natural sciences, mathematics, and language). All first-grade teachers who participated in this study used a synthetic, phonics-based teaching approach. Children were systematically taught all existing grapheme-phoneme relationships and how to derive word pronunciations on the basis of these conversion rules. The average class size in this region of Germany is 21 students. The Hessen area allows for the inclusion of students with special needs into mainstream schools, though special education schools are also available.

#### Italy

Compared with the German pre-primary and primary educational system, the main difference in South Tyrol, Italy, is that, in order to prepare children for reading and writing at school students are provided with plenty of phonological awareness training in kindergarten (Fthenakis, 2008). Students usually begin primary school at the age of 6, but students who were born between January and March and who are still 5 years old when the school year begins may access primary school slightly earlier than their peers. In the South Tyrolian area, there are no specific guidelines for teaching reading and writing in primary school, but the approach used by the teachers who participated in this study was very similar to the approach used by teachers from the Hessen area in Germany and was primarily phonics-based. The average class size in this region of Italy is approximately 16 students. All students, including students with disabilities and students with special educational needs, are included in the standard classes for their respective grades.

### The present study

The purpose of this study is to examine the heterogeneity and prevalence of latent reading profiles among Finnish- and German-reading students in the first and second grades in Finland, Germany, and Italy, using LPA. In addition, this study evaluates the roles of letter-sound connection, phoneme blending, and RAN as they relate to profile membership.

The specific research questions are as follows:

1. What is the heterogeneity and prevalence of each latent profile that can be identified based on the literacy testing (i.e., word-level reading and sentence-level reading) in the first and second grades?
2. What are the roles of letter-sound connection, phoneme blending, and RAN in each student’s reading profile?
3. What roles do children’s age, spoken language (German only vs. German and Italian), and education system (German vs. Italian) play in the heterogeneity and prevalence of latent reading profiles?

## Materials and methods

### Participants and procedure

Three groups of participants, all of whom were starting first grade, took part in this study. The Finnish group consisted of 324 students (153 girls, 171 boys) from 12 different schools and 21 classes in a medium-sized town in central Finland. The majority of students (97.4%) were native Finnish speakers. The mean age of participants at the end of the second grade was 8.91 years (SD 0.58).

The German group consisted of 283 students (123 girls, 160 boys) from seven different schools and 13 classes in a medium-sized town in Hessen. The majority of participants (77.7%) were native German speakers, and 22.3% spoke German and another language. The mean age of participants at the end of the second grade was 8.44 years (SD 0.46).

The Italian group consisted of 162 students (87 girls, 75 boys) from ten different schools and ten classes in South Tyrol. The majority of participants (79%) were native German speakers, and 21% spoke German and another language. The mean age of participants at the end of the second grade was 7.97 years (SD 0.32).

Tests were prepared and administered by native speakers of both targeted languages. Before beginning the study, extensive pilot work was carried out with researchers located in each of the three countries in order to ensure that the testing was appropriate for each context. This was especially important for the phonological awareness tests that were formulated for this study. In all cases, local testers administered the tests so that they, and their accents, would be familiar to the children. Instructions ensured that testers administered the tests in the prescribed manner and in the same order. Before beginning each task, students were given verbal and visual instructions, as well as three examples of the task. The letter-sound connection test was executed in each country exactly 8 weeks after students began the first grade (September or October, depending on each school’s starting date). Word reading (WR1) was measured after 18 weeks (around February, depending on the length of the Christmas holiday in each country), and the second word reading (WR2) and phoneme blending tests were executed after 29 weeks of schooling in each country (around May). In the second grade, the first sentence reading test (SENR1) was executed after 8 weeks (September or October), rapid naming (RAN) was assessed after 18 weeks (around February), and the second sentence reading test (SENR2) was completed after 29 weeks (around May).

At the beginning of the study, the children’s parents and teachers were asked to provide written consent to participate.

### Measures

In Finland, a group-administered subtest, based on the battery of standardized reading tests (ALLU – Reading Test for Primary School) (Lindeman, 1998), was used to assess word-level and sentence-level reading skills. The word-level reading test contained a maximum of 80 words, and students were given no more than 2 min to complete the test. Each test item included a picture and four phonologically similar words, and students were asked to draw a line between the picture and the semantically correct word. The sentence-level reading test (reading comprehension) contained 20 unrelated sentences, and students were given a maximum of 2 min to complete the test. Each item on the sentence-level reading test included a picture and four phonologically and semantically similar sentences, and students were asked to draw a line between the picture and the semantically correct sentence. Two similar versions of each test, A and B, were provided. The variables for word-level reading and sentence-level reading were constructed by calculating the number of correct responses given at each phase (one point was awarded for each correct answer). The Cronbach’s alphas were 0.976 for WR1, 0.973 for WR2, 0.868 for SENR1, and 0.837 for SENR2.

German-reading students’ reading skills were tested using Ein Leseverständnistest für Erst-bis Sechstklässler (A Reading Comprehension Test for First- Through Sixth-Grade Students) (Lenhard & Schneider, 2006). The first subtest assessed each student’s word-level reading accuracy and fluency through picture-word matching. Each of the 72 items on this subtest contained one picture and four written words, and students were asked to identify which word corresponded with each picture. Students were given 3 min to complete as many items as possible. The second subtest measured sentence-level reading and consisted of 28 unrelated sentences, each with one word missing. From a set of five words, the students were asked to identify which word correctly completed each sentence. The students were given 3 min to complete as many items as possible. The variables for word-level reading and sentence-level reading were constructed by calculating the number of correct responses given at each phase (one point was awarded for each correct answer). For the German data, the Cronbach’s alphas were 0.949 for WR1, 0.948 for WR2, 0.894 for SENR1, and 0.908 for SENR2. For the Italian data, the alphas were 0.931 for WR1, 0.944 for WR2, 0.903 for SENR1, and 0.913 for SENR2.

#### Letter-sound connection (LSC)

For the letter-sound connection test, students were given an A4 sheet containing empty boxes. The tester pronounced a phoneme twice, and students were asked to write the corresponding letter in each box, in either upper or lowercase. There are 23 letter sounds in the Finnish language (Holopainen, Mäkihonko, & Bauer, 2010) and 26 letter sounds in the German language (Bauer & Koch, 2010). Cronbach’s alphas were 0.926 for the Finnish data, 0.916 for the German data, and 0.919 for the Italian data.

#### Phoneme blending (PHB)

For the phoneme-blending test, students were given an A4 sheet divided into ten rows, each containing three pictures of common four- to six-letter nouns. A smiley face was printed below each picture. Students were given the following instructions: “Row 1: Listen to the words you hear on the CD. They are pronounced phoneme by phoneme. Each word you will hear corresponds to one of the pictures in the first row. After you have chosen the right picture, color the smiley face underneath that picture.” The total score was calculated based on the sum of correct answers, to a maximum of ten. Cronbach’s alphas were 0.755 for the Finnish test (Holopainen et al., 2010), 0.724 for the German data (Bauer & Koch, 2010), and 0.798 for the Italian data.

#### Rapid automatic naming (RAN)

Two subtests, based on the rapid serial naming test (Dencla & Rudel, 1974), were executed to measure the time it took students to name letters and objects. The sum score of correct answers was standardized for each separate dataset (Finnish and German), as number names vary in length between the two studied languages.

### Data analyses

The skewed variables were first normalized using transformation methods within SPSS (Version 24.0). All continuous variables were then standardized. Although our data was collected from three different countries (Finland, Germany, and Italy), only two languages (Finnish and German) were studied; students in South Tyrol, Italy, learn to read in German. Therefore, the data from Germany and Italy was analyzed as a single German-reading dataset.

In order to answer the research questions, we used the R3step method to analyze each dataset: first, we built several latent profile models; second, after the students had been grouped into different latent reading profiles, the classifications were saved; third, the latent classifications were then related to covariates. To analyze the students’ reading profiles, we estimated a series of LPA models using a different number of classes (mixture modeling method) for both the Finnish and German-reading data, and then we compared the absolute and relative fits of those models. Mplus v.7.4 (Muthén & Muthén, 2015) was used to fit the models. We examined the latent profile models using data from word reading and sentence reading from first and second grade. Different criteria were applied in order to evaluate the LPA model fit and choose the best reading profile models. The criteria were as follows:

1. Information criteria: Akaike Information Criteria (AIC), Bayesian Information Criteria (BIC), and sample size-adjusted Bayesian Information Criteria (aBIC). The model with the smallest value was considered the best choice.
2. Entropy: values close to one indicated good classification accuracy.
3. Statistical testing: parametric bootstrap likelihood ratio difference (BLR), Vuong-Lo-Mendell-Rubin (VLMR), and Lo-Mendell-Rubin adjusted (LMR).

For the relative model fit, significance (*p* values) < .05 indicated that the model with the highest number of profiles was preferred over the model with the lowest number of profiles.

We used ordinal logistic regression modeling to investigate the role of cognitive variables among students’ reading skills across different profiles. Ordinal logistic regression was used to predict an ordinal dependent variable (student reading profiles, in this case), based on one or more predictors or independent variables.

Ordinal logistic regression modeling was also used to investigate the third research question. For this model, students’ reading profiles were selected as the dependent variable, and in Finnish and German reading data age (1 = younger than 6.5 years, 2 = older than 6.51 years), and in German reading data the educational system (German = 1, Italian = 2), spoken language (0 = German only, 1 = German and Italian) were selected as the independent variables.

## Results

### Reading profiles

#### Finnish reading profiles

**Fig. 1:**
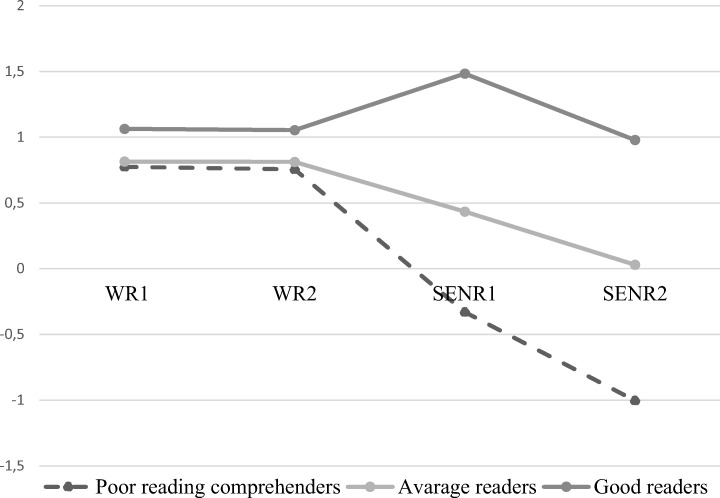
Finnish reading profiles (WR1 = word reading February first grade, WR2 = word reading May first grade, SENR1 = sentence reading September second grade, SENR2 = sentence reading May second grade)

The statistical comparison of reading profile models for Finnish students is shown in Table 1; Fig. 1. Values collected from the information criteria showed that models with more profiles tended to fit the data better than models with fewer profiles. The relative statistical tests, VLMR, LMR, and BLRT, indicated that the three-profile model fit the data better than did other models. Based on the results of the relative model fit tests and the meaningfulness of interpretation, the three-profile model was selected, and the profiles were labeled as poor reading comprehenders, average readers, and good readers.

**Table 1:** Statistical model comparisons for reading profiles of Finnish students (standardized scores)

	Log L	AIC	BIC	Adjusted BIC	Entropy	VLMR	LMR	BLRT	*N* (profile1)	*N* (profile2)	*N* (profile3)	*N* (profile4)	*N* (profile5)	*N* (profile6)
1	− 1247.612	2511.223	2541.370	2515.995					320					
2	− 1158.473	2342.946	2391.934	2350.700	0.709	0.0001	0.0001	0.0000	201	119				
3	− 1110.831	2257.662	2325.492	2268.399	0.791	0.0003	0.0004	0.0000	72	180	68			
4	− 1075.862	2197.724	2284.395	2211.443	0.820	0.3944	0.4049	0.0000	5	106	176	33		
5	− 1040.580	2137.159	2242.672	2153.861	0.798	0.2564	0.2681	0.0000	172	5	58	27	58	
6	− 1015.361	2096.721	2221.076	2116.406	0.774	0.6104	0.6198	0.0000	5	24	53	127	80	31

*Profile 1: Poor reading comprehenders* (proportion: 22.50%). Students in this profile displayed slightly lower word-level reading skills in the first grade compared with students who were classed in other profiles, but their scores were still above-average. In the second grade, as the sentence-level reading tasks became more challenging, the scores among students in Profile 1 fell below the average and were the lowest among all profiles.

*Profile 2: Average readers* (proportion: 53.24%). The reading skills of students in this profile were lower than those of students in Profile 3 but were better than those of students in Profile 1. The above-average scores in word-level reading in the first grade allowed these students to maintain average sentence-level reading skills in the second grade. More than half of all students fell into this reading profile.

*Profile 3: Good readers* (proportion: 21.25%). Students in this profile scored highest on the word-level reading test in the first grade. In second grade, these students continued to maintain the highest sentence-level reading skills among all profiles.

In order to check whether the profiles differed significantly from each other at each indicator level, we preformed Wald’s tests using Profile 3 (good readers) as the referent profile. The results of the tests showed that profiles were significantly different (Wald *X*^*2*^ = 20.763, df = 1, *p* = .000 for poor reader profile, and Wald *X*^*2*^ = 22.451, df = 1, *p* = .000 for average reader profile). Although the tests indicated that profiles did not differ significantly for word-level reading skills (Wald *X*^*2*^ = 0.925, df = 1, *p* = .336 for word reading level 1, and Wald *X*^*2*^ = 0.491, df = 1, *p* = .484 for word reading level 2), they were different for sentence-level reading skills (Wald *X*^*2*^ = 17.789, df = 1, *p* = .000 for sentence reading level 1, and Wald *X*^*2*^ = 22.869, df = 1, *p* = .000 for sentence reading level 2).

#### German reading profiles

**Fig. 2:**
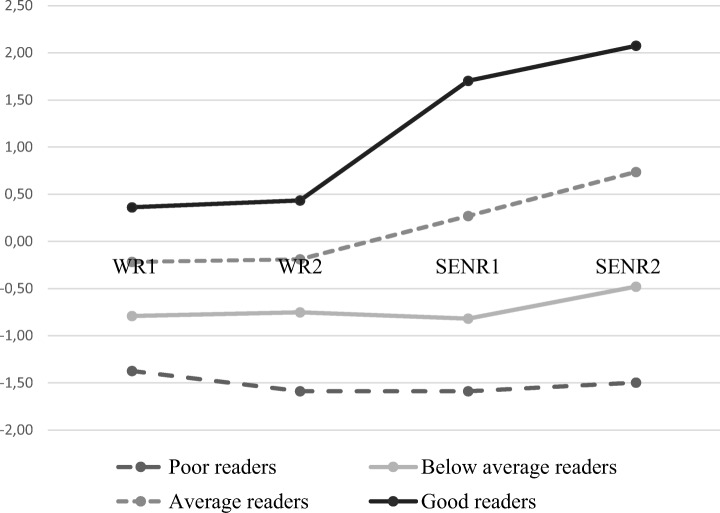
German reading profiles (WR1 = word reading February first grade, WR2 = word reading May first grade, SENR1 = sentence reading September second grade, SENR2 = sentence reading May second grade)

The statistical comparison of four reading profile models for German-reading students is shown in Tables 2, 3, and 4; Fig. 2. Model 4 displayed the lowest BIC value, indicating that this was the preferred model, although the statistical tests (VLMR, LMR, and BLRT) were significant in models 2, 3, and 4. Based on the information criteria and statistical tests, the four-profile model was selected and the profiles were labeled as poor, below-average, average, and good readers.

**Table 2:** Means and standard errors of Finnish reading profiles (standardized scores)

Reading skills	Poor reading profile mean (SE)	Average reading profile mean (SE)	Good reading profiles mean (SE)
Word reading level 1	0.775 (0.116)	0.815 (0.065)	1.064 (0.111)
Word reading level 2	0.756 (0.102)	0.812 (0.062)	1.054 (0.098)
Sentence reading level 1	− 0.330 (0.054)	0.435 (0.059)	1.484 (0.064)
Sentence reading level 2	− 1.003 (0.081)	0.030 (0.045)	0.979 (0.074)

**Table 3:** Statistical model comparisons for reading profiles of German reading students (standardized scores)

	Log L	AIC	BIC	Adjusted BIC	Entropy	VLMR	LMR	BLRT	*N* (profile1)	*N* (profile2)	*N* (profile3)	*N* (profile4)	*N* (profile5)	*N* (profile6)
1	− 2251.937	4521.875	4558.918	4530.355					453					
2	− 1956.783	3943.566	4005.305	3957.700	0.771	0.0000	0.0000	0.0000	254	199				
3	− 1867.148	3776.297	3862.731	3796.084	0.809	0.0028	0.0032	0.0000	193	226	34			
4	− 1787.694	3629.387	3740.516	3654.828	0.794	0.0000	0.0000	0.0000	75	168	182	28		
5	− 1774.152	3614.304	3750.129	3645.398	0.724	0.2713	0.2812	0.0000	61	172	84	112	24	
6	− 1762.086	3602.172	3762.691	3638.919	0.754	0.0209	0.0228	0.0128	169	1	62	25	111	85

**Table 4:** Means and standard errors of German reading profiles (standardized scores)

Reading skills	Poor reading profile mean (SE)	Below-average reading profile mean (SE)	Average reading profile mean (SE)	Good reading profile mean (SE)
Word reading level 1	− 1.374 (0.090)	− 0.791 (0.064)	− 0.218 (0.042)	0.362 (0.099)
Word reading level 2	− 1.588 (0.088)	− 0.751 (0.064)	− 0.190 (0.037)	0.434 (0.080)
Sentence reading level 1	− 1.589 (0.091)	− 0.819 (0.068)	0.269 (0.075)	1.703 (0.200)
Sentence reading level 2	− 1.498 (0.118)	− 0.479 (0.092)	0.737 (0.079)	2.074 (0.120)

*Profile 1: Poor readers* (proportion: 16.55%). Students in Profile 1 seemed to be the most challenged group of readers among the German-reading data, with both word-level and sentence-level reading skills measuring approximately 1.5 z-scores below the mean level. Compared with other groups, the development of reading skills among students in Profile 1 appeared to be very slow.

*Profile 2: Below-average readers* (Proportion: 37.09%). Students in Profile 2 exhibited higher reading levels than did students in Profile 1 but exhibited lower reading levels than did students in Profiles 3 and 4. Students in Profile 2 also demonstrated slowed reading development during their first 2 years of schooling.

*Profile 3: Average readers* (proportion: 40.18%). Most students belonged to Profile 3. In the first grade, students in this profile displayed slightly below-average reading skills; however, in the second grade, their sentence-level reading skills were above-average, showing good development of reading skills.

*Profile 4: Good readers* (proportion: 6.79%). In the first grade, students in this profile scored above-average and received the highest scores of all profiles. In the second grade, they continued to improve their reading abilities and maintained the highest level of reading skills.

The results of Wald’s testing among the German-reading group showed that the profiles were significantly different from each other at all indicator levels, including word-level (Wald *X*^*2*^ = 11.112, df = 1, *p* = .001 for word reading level 1, and Wald *X*^*2*^ = 17.211, df = 1, *p* = .000 for word reading level 2) and sentence-level reading skills (Wald *X*^*2*^ = 13.427, df = 1, *p* = .000 for sentence reading level 1, and Wald *X*^*2*^ = 18.889, df = 1, *p* = .000 for sentence reading level 2).

### Relationship between cognitive variables and students’ reading profiles

The results of the ordinal logistic regression analysis showed that the final models, which included predictor variables, improved upon the intercept-only models for both Finnish and German-reading data.

For the Finnish data, the model fitting information was: − 2 log likelihood = 426.380 (intercept-only model), 401.425 (final model); X^2^ = 24.955, df = 3, *p* = .000. The analysis showed that RAN was significantly related to students’ reading profiles (see Table 5). Students who completed the naming tests quickly were more likely to belong to a better reading profile. If a student scored one unit higher in RAN (took more time to complete the naming test), that student’s ordered log-odds of being classed in a higher reading profile decreased by − 0.634, while other model variables remained constant. Letter-sound connection and phoneme blending were not significantly related to students’ reading profile memberships.

**Table 5:** Impacts of letter-sound connection, phoneme blending, and rapid automatic naming on Finnish and German reading profiles

Reading profiles		B	Std. error	Sig.
Finnish reading profiles	Letter-sound connection	0.218	0.193	0.257
	Phoneme blending	− 0.057	0.153	0.710
	Rapid automatic naming	− 0.634	0.136	0.000
German reading profiles	Letter-sound connection	0.411	0.096	0.000
	Phoneme blending	0.128	0.101	0.206
	Rapid automatic naming	− 0.718	0.109	0.000

For the German-reading data, the model fitting information was − 2 log likelihood = 829.569 (intercept-only model), 746.937 (final model); X^2^ = 82.632, df = 3, *p* = .000. Letter-sound connection and RAN were significantly related to students’ reading profiles. Students who exhibited better letter-sound knowledge were more likely to be classed in a higher reading profile. Similar to the Finnish data, German-reading students who were classed in higher reading profiles were more likely to perform faster on naming tests and better on letter-sound connection tests. If a student scored one unit higher on the letter-sound connection test, that student’s odds of being classed in a higher reading profile increased by 0.411, while other model variables remained constant. Phoneme blending was not significantly related to students’ reading profiles (see Table 5).

### Relationship of child’s age, spoken language, education system, and reading profiles

The results of ordinal logistic regression modeling, using reading profiles as the dependent variable and educational system, child’s age, and child’s spoken language (only German or German and other language) as the independent variables, showed that the educational system (German or Italian) was not significantly related to students’ reading profiles (B = − 0.080, S.E. = 0.082, *p* = .332). A child’s age and spoken language were also not significantly associated with their reading profile for the child’s age, B = − 0.028, S.E. = 0.274, *p* = .920; for the child’s spoken language, B = 0.203, S.E. = 0.300, *p* = .499).

## Discussion

As several previous cross-linguistic comparisons have shown the accurate and relatively fast development of decoding skills among learners of orthographically transparent languages (e.g., Landerl et al., 2013; Rothe, Schulte-Körne, & Ise, 2014; Seymour et al., 2003), we wanted to expose the latent reading groups that were prevalent among students who were learning to read in two separate transparent languages, German and Finnish. Through this person-oriented analysis, the aim was not only to show the possible orthographical differences in reading development but also to add to the existing knowledge about the possible differences in word-level and sentence-level reading skills and to explore the relationships between reading-related variables and profile membership. Additionally, as children in these three countries enter school at different ages, we wanted to analyze the effect of children’s age as an independent variable in our modeling. Moreover, in the German reading data, there was a group of bilingual students speaking German and another language, and because the education systems in Germany and Italy have some differences, we analyzed the role of spoken language and educational system in the German reading data.

As part of this study, we analyzed the word- and sentence-level reading skills of 769 students in the first and second grades. The results indicate that, in the first and second grades, three latent profiles appear among Finnish students, and four latent profiles appear among German-reading students. Slowed RAN timing among both datasets, as well as poorer letter-sound connection results among German-reading students, was significantly related to poorer reading profiles. Interestingly, the German and Italian educational systems had no significant effect on the students’ profiles.

Among transparent orthographies, letter-sound connection is very clear, and words are generally pronounced as they are written (e.g., Rothe et al., 2014; Vellutino et al., 2004). On average, compared with opaque orthographies, students who learn to read in transparent orthographies have a greater advantage and tend to develop their word-level reading skills much faster. In this study, as predicted by the orthographic depth hypothesis (Katz & Frost, 1992), this advantage was most noticeable in the latent word-level reading profiles of Finnish students, where the students’ word-level reading skills were already quite high by the middle of first grade across all latent profiles (21% of students were classed as good readers, while 53% of students were classed as average readers). The fact that Finnish students begin their formal education at the age of seven, while German and Italian students begin school at the age of six, may also have a positive effect on the development of reading skills among Finnish students in the first and second grades, as their linguistic awareness may generally be more advanced. Moreover, the focus on phonological awareness and letter-sounds practice in pre-primary education may help students learn to read more fluently (Hulme et al., 2012; Kirby et al., 2003; Landerl et al., 2013).

Among German-reading students, only approximately 7% achieved good word-level reading skills after half a year of schooling; thus, 40% of German-reading students were classed as average readers. Compared with the relative simplicity of Finnish orthography, there are more phonemes in the German language, and graphemes can consist of one, two, or three letters. This difference means that children who exhibit poorer reading skills typically know the letter sounds but are unable to blend the sounds (e.g., *strolchst*) into syllables (Landerl & Wimmer, 2008). Moreover, the German language has many complex consonant clusters in both the onset and coda positions, and word stems can be spelled the same way across different morphophonological contexts. Morpheme knowledge plays an important role in fluent word identification and, especially, in sentence-level reading and reading comprehension (Ziegler et al., 2003).

In this study, the administered word-level and sentence-level reading tests for both orthographies were time-limited and measured both reading accuracy and fluency. Problems with reading fluency have been highlighted in previous studies as one challenge faced by poor readers, especially in transparent orthographies. Problems with reading fluency also appear to be quite permanent (e.g., Landerl & Wimmer, 2008; Lyytinen et al., 2005). The language-specific sentence-level reading challenges faced by Finnish students can be seen in this study among Finnish Profiles 1 (poor reading comprehenders) and 2 (average readers). The Finnish language is a strongly inclined and agglutinating language, with very long written words that are each comprised of a vast amount of semantic and morphological information (Karlsson, 2008). The task used to test students’ sentence-level reading skills in this study also challenged students’ reading comprehension skills, as they were asked to choose one of four similar sentences to match the picture in question. Good word-level reading skills do not help all readers maintain or improve their sentence-level reading abilities or fluency, which can be seen in the clear distribution of students between Profiles 3 (good readers) and 1 (poor reading comprehenders). For students who exhibit slowed reading abilities—although their word-level reading accuracy is average—there can be fewer cognitive and linguistic resources available for higher-level processes of text integration and comprehension not even sentence-level (Cain, 2009; Nation, 2008; Oakhill, Cain, & Bryant, 2003). Additionally, based on the cognitive variables, RAN appears to be related to poorer reading levels in both languages, as seen in many previous studies (e.g., Ozernov-Palchik et al., 2017; Vellutino et al., 2004; Wimmer, 1996).

Problems with sentence-level reading were also apparent among German-reading students, especially those classed in Profiles 1 (poor readers) and 2 (below-average readers), and very little reading improvement was seen after the first grade. The same negative reading comprehension growth pattern was established in a study of students in third through seventh grades that was conducted by Schulte, Stevens, Elliott, Tindal, and Nese (2016), and again in a study of children aged 7 through 17 that was conducted by Wei, Blackorby, and Schiller (2011). Both studies concluded that the developmental reading comprehension course for students with disabilities can be defined as a deficit model, in which the initial differences in skill levels persist over time with no sign of lower-achieving groups catching up to higher-achieving groups.

German-reading students who were classed in Profiles 3 (average readers) and 4 (good readers) were able to further use their decoding skills for reading comprehension and managed both reading tasks well in the second grade. The sentence-level reading task administered to both Finnish and German-reading students, although a bit different, required good reading fluency and comprehension skills. Slower RAN test results were also related to poorer reading profiles among both Finnish and German-reading students. In addition, problems with letter-sound connection, which was measured halfway through the school year, were significantly related to poorer reading profiles, illustrating the importance of intensive letter-sound practice before children begin school.

We also analyzed the role of the educational system in German-reading students’ profiles, as the educational systems in Germany and South Tyrol, Italy, have some differences. However, the different countries’ educational systems did not play a significant role in determining the German reading students’ reading profiles. This result might be partially related to the fact that, in all three countries evaluated in this study, students attend pre-primary education prior to beginning formal schooling, and the method of reading instruction employed during the first school year, for all schools that participated in this study, was phonics-based, which should boost students’ acquisition of phonological recoding skills and help students to develop word-level reading skills (Ziegler & Goswami, 2006).

## Limitations

There are some limitations that should be considered when generalizing the results of this study. First, although the study’s sample size was relatively large (almost 770 students), the sample was selected from only one area of each country. Second, for this study, instead of using individual tests, we used group-administered tests to measure reading, letter-sound knowledge and phoneme blending, and due to limited resources, no vocabulary or other linguistic measures (e.g., measures on morphology) were used. Third, although we administered similar tests in each country, differences between these tests might have influenced the results. Fourth, as previous studies have shown that parents’ good educational level has positive effects on their children’s reading skills, it would have been important to add SES as a covariance to the models. In this study, we have information from parents’ SES, but unfortunately, the missing information is quite large (58.7% for fathers and 57.8% for mothers); therefore, we could not include the SES in the analysis.

## Conclusion and implications

Our findings favor the view that, when using a person-oriented approach, reading difficulties that appear within two transparent orthographies can manifest quite differently in word-level and sentence-level reading skills among students in the first and second grades. The results show that, after half a year of schooling, there appear to be two profiles of slowly developing reading skills among the German-reading group and one profile of slowly developing reading skills among the Finnish group. It is very important to identify students who are not able to apply their word-level reading skills to their text and reading comprehension skills in order to develop proper support systems for these students.

Despite the limitations of this study, we believe that our research findings have practical implications for educators who are working to teach and improve students’ reading skills. Early identification of slow reading progress is essential, as early and intensive intervention is needed in order to change the negative reading trajectories of students with disabilities (Fuchs, Fuchs, & Vaughn, 2014). Moreover, teachers require information regarding the results of different interventions in order to apply evidence-based individual reading interventions (Lemons, Al Otaiba, Conway, & Mellado De La Cruz, 2016); as Vaughn & Fletcher, (2012) and Allor et al. (2010) state, “one-size-fits-all” interventions are not effective methods of supporting different risk profiles. As illustrated by the summary of research that was conducted between 2004 and 2014 on interventions designed to support reading fluency among elementary students, the increase in reading fluency that occurs with fluency-based instruction is often associated with better reading comprehension outcomes (Kim, Bryant, Bryant, & Park, 2017). In addition, Wanzek, Wexler, Vaughn, and Ciullo (2010) suggest that elementary students who exhibit reading and learning difficulties might benefit from fluency-based interventions that address word recognition.

The goal of our next study may be to discover which types of language-specific support are provided to students in low-performing profiles in two languages (German and Finnish), and which other cognitive (e.g., IQ, working memory) and background (e.g., SES) variables might explain students’ profile memberships. These findings are important, given the large number of low-performing readers in many countries and the limited time and resources available to educators.
